# From Sensor Data to Educational Insights

**DOI:** 10.3390/s22218556

**Published:** 2022-11-07

**Authors:** José A. Ruipérez-Valiente, Roberto Martínez-Maldonado, Daniele Di Mitri, Jan Schneider

**Affiliations:** 1Department of Information and Communication Engineering, University of Murcia, 30100 Murcia, Spain; 2Faculty of Information Technology, Monash University, Clayton, VIC 3168, Australia; 3DIPF|Leibniz Institute for Research and Information in Education, 60323 Frankfurt, Germany

Technology is gradually becoming an integral part of learning at all levels of educational. This includes the now pervasive presence of virtual learning environments (VLEs) and the inclusion of interactive devices used or worn by learners or that are present in the physical classroom environment. These new technology-rich educational ecosystems have greatly facilitated data capture about learners. Thus, several research areas, such as learning analytics (LA), educational data mining (EDM), and artificial intelligence in education (AIED), have grown exponentially during the last decade, with multiple venues supporting this research [1]. However, the inferences about learning that can be made by solely analyzing trace data from VLEs are limited, since logged data do not commonly provide a complete view of the learning experience [2]. Therefore, research communities are moving beyond the data obtained from VLEs and other online tools by incorporating data from external sources such as sensors, pervasive devices, and computer vision systems. Within the context of education, this subfield is often denominated as multimodal learning analytics (MMLA) [3]; nevertheless, the use of these data sources is also common in broader research areas, such as affective computing (e.g., [4]) and human–computer interaction (HCI) (e.g., [5]). The promise is to augment and improve the extent and quality of the analysis that can be performed with these new data sources [6]. Moreover, many new sensor-based tools, such as sensor-based games [7] or realistic laboratories [8,9], are being built to support the educational process. The challenge is embedding sensors and resulting data representations in authentic educational settings in pedagogically meaningful and ethical ways [10].

This Special Issue (SI) invited publications that include approaches to converting data captured using sensors (e.g., cameras, smartphones, microphones, or temperature sensors), wearables (e.g., smart wristbands, watches, or glasses), or other Internet of Things (IoT) devices (e.g., interactive whiteboards, eBooks, or tablets) into meaningful educational insights. Moreover, it invited papers on tools, architectures, or frameworks to manage the orchestration of these sensors and IoT devices to improve education. The submitted articles had to appropriately explain how the inclusiveness of sensor devices can augment the analyses performed to improve teaching, learning, or the educational context in which the sensing it occurs (e.g., in classrooms, VLEs, or other educational spaces). This SI has focused on empirical case studies that fulfill the aforementioned criteria and experimental architectures, methodologies, frameworks, or survey papers.

## 1. The Affordances and Caveats of Sensor Data in Education

Using sensor data in education offers researchers novel perceived affordances to generate a richer picture of the learning experience by going beyond what can be captured from mouse clicks and keystrokes. Sensor data can enable the automated analysis and support of learning activities that are not necessarily mediated by a computer [11], such as activity unfolding in a maker space [12] or in physical simulation-based training rooms [13]. Similarly, video, audio devices, and other sensing devices have been used in physical learning spaces to model aspects of the classroom that, in the past, could only be studied via direct observations and ethnographic studies such as teacher–student communication [14], spatial dynamics [15], and students’ collaborative dialogue [16]. Data obtained from both wearables and video-based body part recognition algorithms are also enabling the study of learning activities that involve the development of psychomotor skills, such as the effective performance of cardiovascular resuscitation for the case of clinical students [17] or the delivery of oral presentations by effectively combining gestures and body posture [18]. Sensor data are also being used to generate a deeper understanding of *under-the-skin* cognitive and emotional aspects of the student that may affect learning but that are hard to perceive without sensing devices [19].

However, this increased interest in using sensor data in education comes with some important caveats. The risks of unintended over-surveillance and the capture of activities that are not directly related to learning need to be addressed [20]. For example, using video in learning spaces can bring numerous ethical implications since not all the activities that occur in a classroom may be related to the subject matter being learned, and students can be easily identified [21]. The use of physiological sensors can also generate information that can be considered private and personal by many learners [22]. Moreover, another issue involves the enlarged amount and heterogeneity of data being captured, which also increases the complexity of the learning analytics solution. Whilst it may be possible to find interesting trends from the data and provide more accurate learning models, the intricacy of the models and the sensing setup may make the solution harder to implement under authentic learning situations [3]. It may also become challenging to translate low-level sensor signals into meaningful educational constructs that teachers and students can understand [6,13]. This can also make full consenting from the perspective of students, educators and other educational stakeholders more challenging, as it may be harder for them to fully understand the implications of capturing and analyzing each data modality [11,23]. Together, the added complexity in infrastructure and expertise required and informed consenting may threaten the long-term sustainability and scalability of sensor-based educational solutions, which has already been identified in a recent MMLA literature review [10]. In sum, much research is needed to develop sensor-based MMLA systems with integrity that balance the benefits of augmenting the learning situations with the potential ethical and practical challenges that this conveys. This SI contributes in several aspects to these open issues and research gaps.

## 2. Overview of the Special Issue

The SI has gathered 12 articles on diverse topics that have used sensors within educational environments. Four main uses of sensors in education can be identified in these articles. First, there are articles evaluating sensor-based tools exclusively designed for educational purposes. Some articles studied the use of sensor data to improve the learning process directly. Next, some articles consisted of case studies that collected sensor data to provide new insights into the learning process. Finally, we identified articles with diverse objectives. We will now review the articles of the SI in thematic groups.

First, there is a group of three papers that have implemented tools based on sensors for educational purposes. Guerrero-Osuna et al. [8] presented the design and implementation of a novel IoT device called MEIoT weather station. This tool can be used as a hybrid educational IoT environment. They presented a case study regarding how to use it to teach the least-squares regression topics for linear, quadratic, and cubic approximations within the Educational Mechatronics Conceptual Framework (EMCF). Another example is the article published by Khan et al. [7], who proposed a 3D realistic open-ended VR and Kinect sensor-based training setup using the Unity game engine, wherein children are educated and involved in road safety exercises. The sensors of the game make the game experience much more immersive, and the experimental results reported positive outcomes, encouraging good behaviors of the children in terms of the road crossing. Last, the third article related to these topics implemented a Two-Dimensional Cartesian Coordinate System Educational Toolkit (2D-CACSET) to teach the two-dimensional representations as the first step to construct spatial thinking using multiple sensors [9].

Then, another group of three articles implemented applications that use data from sensors to potentially improve the learning process. For example, Mat Sanusi et al. [24] designed and implemented the Table Tennis Tutor (T3), a multi-sensor system consisting of a smartphone device with built-in sensors for collecting motion data and a Microsoft Kinect for tracking body position that could be used to perform live coaching and feedback of the table tennis forehand strokes of the trainee. Then, the work of [25] explored the factors from the physical learning environment (PLE) that can affect distance learning and built a software infrastructure that can measure, collect, and process the identified multimodal data from and about the PLE by utilizing mobile sensing. Finally, they conducted an evaluation with 10 participants regarding what extent the software can provide relevant information about the learning context. The last article of this group by Praharaj et al. [26] prototyped a tool that can perform automatic collaboration analytics using both non-verbal and verbal audio indicators. This tool could be used in collaborative learning activities to better understand the actions and discourse of each member during the activity.

In addition, a group of three articles focused on using sensor data to understand better the learning process within specific case studies and contexts. Two of these articles have focused on the eye gaze of students. The article by Lee et al. [27] used Tobii Pro Glasses 2 to capture eye gaze and developed eye movement analysis with hidden Markov models (EMHMM) to differentiate between the states of focused attention and mind-wandering. They found that participants with the centralized pattern had better performance detecting targets and rated themselves as more focused than those with the distributed pattern, highlighting eye movement patterns differences between attention states (focused attention vs. mind-wandering). A second study by Brückner et al. [28] also focused on eye gaze, but in this case, they used Epistemic Network Analysis (ENA) within the context of graph tasks. They found differences between the gaze patterns of students who solved the graph tasks correctly and incorrectly; for example, incorrect solvers shifted their gaze from the graph to the *x*-axis and from the question to the graph comparatively more often than correct solvers. The last article of this group by Vujovic et al. [29] used collected audiovisual data, within a collaborative activity in a co-located physical learning space, to explore the effects of table shape on collaboration when different group sizes and genders are considered. They found that table shape influences student behavior when considering different group sizes and genders.

Finally, we have the last group of three additional papers that have diverse objectives in their work. For example, Solé-Beteta et al. [30] proposed a methodology and associated model to measure student engagement in VLEs using more than 30 digital interactions and events during a synchronous lesson. Of course, many of these digital interactions are captured via sensors, such as students’ faces, gestural poses, or even audio from their voices. They also validated this methodology by building a software prototype tested in two different synchronous learning activities. The article by [31] presents an evaluation framework to assess the generalizability of machine learning models that use sensor data for LA called EFAR-MMLA and tests it with two datasets with audio and log data; the authors concluded that the framework does indeed help to solve the problematic issue of generalizability. Finally, the last paper Horvers et al. [32] conducted a systematic literature review on how the electrodermal activity (EDA), collected by sensors, is currently used within learning settings. They screened more than 1200 records to finally keep 27 studies in the review, finding considerable variation in the usage of EDA and inconsistent associations between physiological arousal and learning outcomes. They concluded that there is a need for explicit guidelines and standards for EDA processing in educational contexts due to the variability found in the review.

All these SI articles are open access and accessible through the following link: https://www.mdpi.com/journal/sensors/special_issues/sdei (Last access date: 1 November 2022).

## 3. Conclusions and Future of Sensor-Based Technologies in Education

The SI has gathered diverse papers demonstrating the affordances of using sensor data or sensor-based tools within educational settings. However, we also need to consider the potential caveats and drawbacks within this research area to mitigate the risks while optimizing the benefits. Although numerous studies are emerging in the literature, it is still challenging to find practitioners using these technologies in day-to-day teaching. Therefore, it is hard to find significant findings that are robust enough to identify underlying dependencies across the explored variables or to replicate studies to see whether results are universal and which of them are context-dependent [10]. We need more resilient science in this context that can help overcome these current limitations.

The future of sensors and multimodal innovations in education is promising, and there are certain directions where it can have a real positive impact. For example, to improve the training of professionals that need to develop complex cognitive abilities that are required for a role (such as stress regulation in works under heavy pressure [33]) or those that require practicing specific motor skills (such as nurses or doctors in healthcare education [5]). Moreover, these technologies can also be promising to support remote teaching and distance learning with more realistic and immersive activities that more closely mimic face-to-face embodied learning experiences while maintaining the flexibility of these learning modalities [34]. However, future work should also tackle some main challenges, such as model transfer across contexts, ethical and equity concerns, scalability, and good alignment with the instructional design, among many others.

## References

## Data Availability

Not applicable.
